# Stability of toxin gene proportion in red-pigmented populations of the cyanobacterium *Planktothrix *during 29 years of re-oligotrophication of Lake Zürich

**DOI:** 10.1186/1741-7007-10-100

**Published:** 2012-12-07

**Authors:** Veronika Ostermaier, Ferdinand Schanz, Oliver Köster, Rainer Kurmayer

**Affiliations:** 1 University of Innsbruck, Research Institute for Limnology, Mondseestrasse 9, 5310 Mondsee, Austria; 2 Limnological Station, Institute of Plant Biology, University of Zürich, Seestrasse 187, CH-8802 Kilchberg, Switzerland; 3 Zürich Water Supply, Hardhof 9, 8021 Zürich, Switzerland

**Keywords:** allelic discrimination assay, eutrophication, genetic population structure, harmful algal blooms, historic samples, long-term monitoring, microcystin

## Abstract

**Background:**

Harmful algal blooms deteriorate the services of aquatic ecosystems. They are often formed by cyanobacteria composed of genotypes able to produce a certain toxin, for example, the hepatotoxin microcystin (MC), but also of nontoxic genotypes that either carry mutations in the genes encoding toxin synthesis or that lost those genes during evolution. In general, cyanobacterial blooms are favored by eutrophication. Very little is known about the stability of the toxic/nontoxic genotype composition during trophic change.

**Results:**

Archived samples of preserved phytoplankton on filters from aquatic ecosystems that underwent changes in the trophic state provide a so far unrealized possibility to analyze the response of toxic/nontoxic genotype composition to the environment. During a period of 29 years of re-oligotrophication of the deep, physically stratified Lake Zürich (1980 to 2008), the population of the stratifying cyanobacterium *Planktothrix *was at a minimum during the most eutrophic years (1980 to 1984), but increased and dominated the phytoplankton during the past two decades. Quantitative polymerase chain reaction revealed that during the whole observation period the proportion of the toxic genotype was strikingly stable, that is, close to 100%. Inactive MC genotypes carrying mutations within the MC synthesis genes never became abundant. Unexpectedly, a nontoxic genotype, which lost its MC genes during evolution, and which could be shown to be dominant under eutrophic conditions in shallow polymictic lakes, also co-occurred in Lake Zürich but was never abundant. As it is most likely that this nontoxic genotype contains relatively weak gas vesicles unable to withstand the high water pressure in deep lakes, it is concluded that regular deep mixing selectively reduced its abundance through the destruction of gas vesicles.

**Conclusions:**

The stability in toxic genotype dominance gives evidence for the adaptation to deep mixing of a genotype that retained the MC gene cluster during evolution. Such a long-term dominance of a toxic genotype draws attention to the need to integrate phylogenetics into ecological research as well as ecosystem management.

## Background

There is growing concern about observations of harmful algae blooms promoted by nutrient enrichment both in freshwater and estuarine systems. The dominant species frequently include bloom-forming cyanobacteria such as the genera *Anabaena*, *Microcystis *and *Planktothrix*, whose growth may also be favored by elevated temperature [1]. However, efforts of lake restoration, such as the reduction of nutrient input, can also lead to the proliferation of stratifying cyanobacteria such as *P*. *rubescens *because of an increased underwater light regime [2].

It is widely agreed that the production of microcystin (MC), which is the most abundant toxin in freshwater, is directly related to the cell division rate of a particular isolate grown under controlled laboratory conditions [3]. In nature, blooms of cyanobacteria are typically composed of toxic and nontoxic genotypes, the latter resulting from the loss [4] or the inactivation of the MC synthetase (*mcy*) gene cluster [5,6]. Some indices show that the production of MC has a selective advantage for the producers [7]. However, besides the fact that MC is a potent inhibitor of eukaryotic protein phosphatases 1 and 2A, the cellular function of MC is not known. To elucidate parameters that influence the competitive ability of toxic and nontoxic genotypes, several growth experiments with toxic and nontoxic strains of *Microcystis *or *Planktothrix *have been performed under controlled laboratory conditions. Vézie *et al. *[8] reported that under high nutrient levels (nitrogen, phosphorus) toxic strains of *Microcystis *grew faster than nontoxic strains, while Briand *et al. *[9] found an advantage of toxic over nontoxic *Planktothrix *strains when environmental conditions limited growth (through dim light, low temperature or nitrogen-limiting conditions). Furthermore, toxic *Microcystis *strains were shown to be the better competitor at high irradiances [10] when compared with nontoxic strains. The rather contrasting conclusions obtained from different toxic and nontoxic strains possibly result from physiological adaptations of the individual genotypes to specific environmental conditions, which are not related to MC production. Indeed, it has been shown that, within the genus *Planktothrix*, the origin of nontoxic strains is rather ancient, and that toxic and nontoxic strains evolved independently and differentiated physiologically in response to environmental factors not directly related to toxin production [4]. Only one monophyletic lineage of green-pigmented strains of the genus *Planktothrix *that lost the *mcy *gene cluster (henceforth referred to as lineage 1) could be found, which invaded shallow, polymictic lakes throughout Europe [4]. A second lineage containing both red- and green-pigmented strains (henceforth referred to as lineage 2) retained the *mcy *gene cluster. This phylogenetic evidence can explain why red-pigmented (phycoerythrin-rich) populations of *Planktothrix*, which typically occur in deep, stratified lakes and reservoirs, are commonly composed solely of the genotype containing the *mcy *gene cluster [5,11]. By contrast, green-pigmented populations that dominate in shallow, eutrophic and polymictic water bodies have a much higher proportion of the nontoxic genotype. A recent survey on toxic genotype abundance in European lakes revealed that red-pigmented populations of *Planktothrix *show a significantly higher proportion of the toxic genotype when compared with green-pigmented populations [12].

Only few studies investigated the selective advantage of red- versus green-pigmented *Planktothrix *ecotypes under field conditions. Davis *et al. *[13] investigated a mixed-pigmented *Planktothrix *population in Blelham Tarn, Lake District, England and analyzed the vertical distribution of the biomass of the two ecotypes. For both ecotypes, the biovolume was increasing under stratified conditions of the water column. However, the red-pigmented ecotype could be shown to grow at greater depths under stratifying and mixed conditions, as its compensation light intensity for growth was lower compared with the green-pigmented ecotype. It is known that *P. rubescens *is adapted to low light conditions whereas *P. agardhii *is more tolerant to high light intensities [14,15]. Oberhaus *et al. *[15] suggested further that the combined effects of temperature and light quality and quantity influence the proliferation of *P. rubescens *and *P. agardhii*. They found the red-pigmented strain to be more competitive at lower temperatures (15°C) and low intensities of green light, resembling the conditions present in the metalimnion, whereas the green-pigmented strain was more competitive at higher temperatures (25°C) and generally less specialized to light quality. Similarly, Stomp and colleagues [16] showed that the underwater light regime was an important factor for niche differentiation of red- and green-pigmented picocyanobacteria and reported their coexistence in waters of intermediate turbidity, whereas red-pigmented picocyanobacteria dominated in clear waters and green-pigmented picocyanobacteria were dominant in turbid waters. Additionally, Walsby and co-workers [17] suggested that the resistance of gas vesicles against hydrostatic pressure is of major importance during lake mixing, especially in deep lakes when filaments become entrained in the hypolimnion. A selective difference between red- and green-pigmented strains producing different types of gas vesicles has been suggested [18], which could further influence the dominance of the red-pigmented ecotype in deep habitats and the common abundance of the green-pigmented ecotype in more shallow water bodies. However, still not much is known about the temporal stability of those contrasting ecotype strategies in ecosystems subject to severe shifts in local environmental conditions.

Here, we report the detailed analysis of the genotypic population structure of *Planktothrix *spp. in Lake Zürich, Switzerland, covering a time span of almost 30 years, which was facilitated by the isolation of DNA from phytoplankton preserved on filters. Because Lake Zürich represents an important drinking water source for about 900,000 inhabitants, its planktonic phytoplankton composition has been monitored intensively. Lake Zürich underwent a well-documented history of eutrophication that reached its maximum around 1965. The re-oligotrophication process was initiated by reducing the input of phosphorus [19]. Except for the period of maximum eutrophy (1965 to 1975), *Planktothrix *occurred in Lake Zürich during the whole century. The first *Planktothrix *bloom was recorded in 1897. During the eutrophic period, eukaryotic algae frequently formed surface blooms, which subsequently disappeared as a result of re-oligotrophication measures, while *Planktothrix *consistently increased [20].

The aim of the study was to find out whether (i) the abundance of the toxic *Planktothrix *genotype changed during the observation period, that is from the period with minimum population density and almost complete disappearance to a stable dominance of the phytoplankton community; (ii) nontoxic genotypes (including inactive mutants) increased in proportion during the observation period: following the hypothesis of Briand *et al. *[21] we would expect an increase of nontoxic genotypes parallel to the increase of the total population density; (iii) the green-pigmented ecotype was present and was of selective advantage under certain environmental conditions, for example at the beginning of the 1980s when the euphotic zone was rather shallow and the red-pigmented ecotype was disfavored because of the high absorption coefficient in the water column.

## Results

### Long-term changes in phytoplankton composition in Lake Zürich

**Figure 1:**
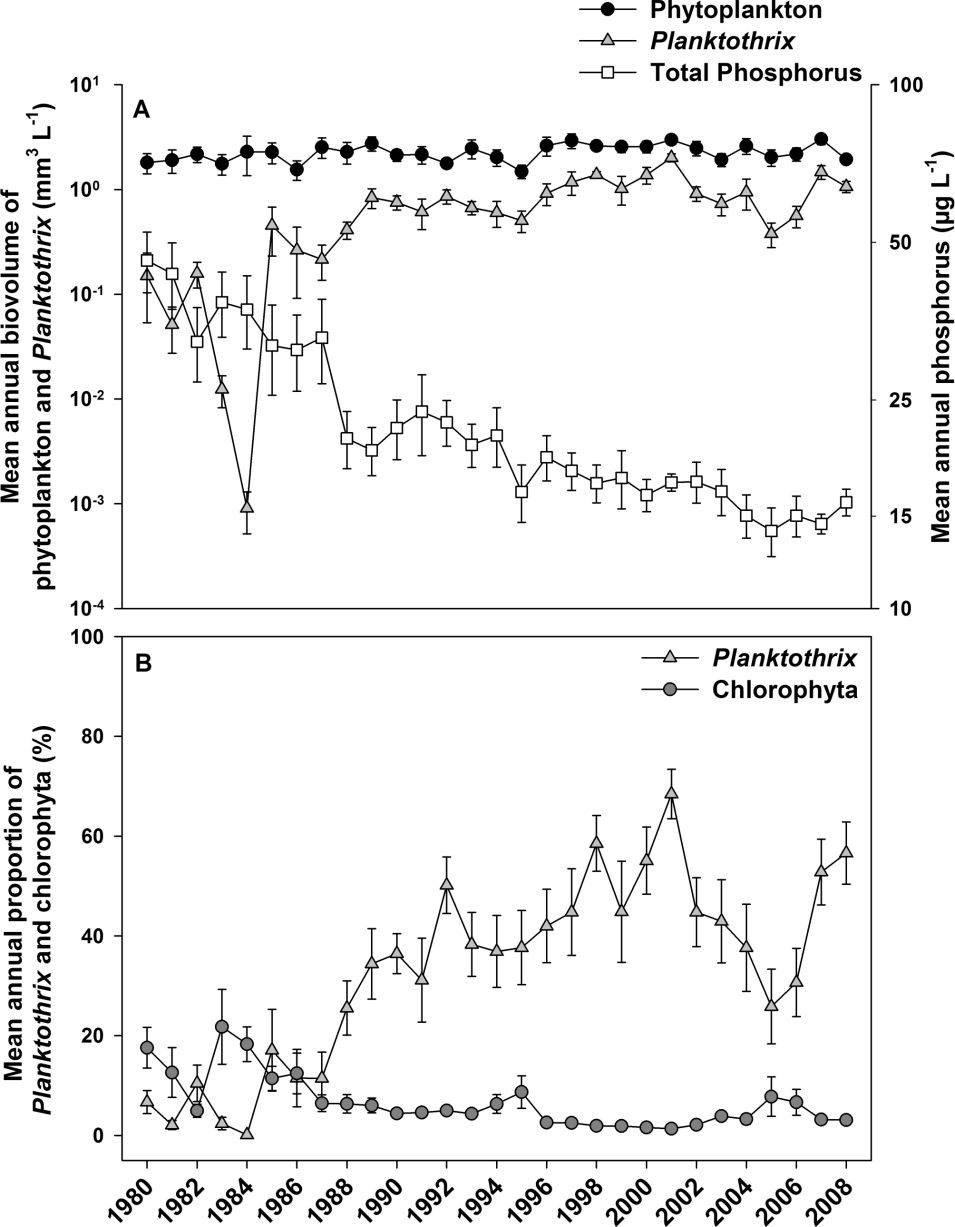
**History of phytoplankton and total phosphorus concentrations between 1980 and 2008**. (A) Annual mean ± SE biovolume of total phytoplankton, *Planktothrix *and concentration of total phosphorus in Lake Zürich from 1980 to 2008. Biovolume in mm^3 ^L^-1^, determined by microscopic counting, total phosphorus in μg L^-1^. (B) Annual mean ± SE percentage of *Planktothrix *and chlorophyta out of total phytoplankton.

Although the mean total phytoplankton biovolume per year showed little change between 1980 and 2008 (minimum 1.5 mm^3 ^L^-1^, maximum 3.0 mm^3 ^L^-1^, mean 2.3 ± 0.1 mm^3 ^L^-1^), the mean *Planktothrix *biovolume per year underwent pronounced fluctuation (Figure 1). During a population collapse in 1984, with a minimum of 0.001 mm^3 ^L^-1^, *Planktothrix *accounted for only 0.1% of the total phytoplankton and 6.0% of the total cyanobacterial biovolume. Subsequently, the population recovered and increased up to a maximum of 2.0 ± 0.2 mm^3 ^L^-1 ^in 2001. Within the period between 1985 and 2008, *Planktothrix *contributed on average 39 ± 2.9% to the total phytoplankton (minimum 11.5%, maximum 68.4%) and 87 ± 2.8% to the total cyanobacterial biovolume (minimum 47.6%, maximum 98.2%). Excepting 2005, the share of *Planktothrix *of the total phytoplankton exceeded 70% in the period from August to March during the years 1995 to 2008 (*n *= 43).

### Quantification of toxic *Planktothrix *in Lake Zürich

**Figure 2:**
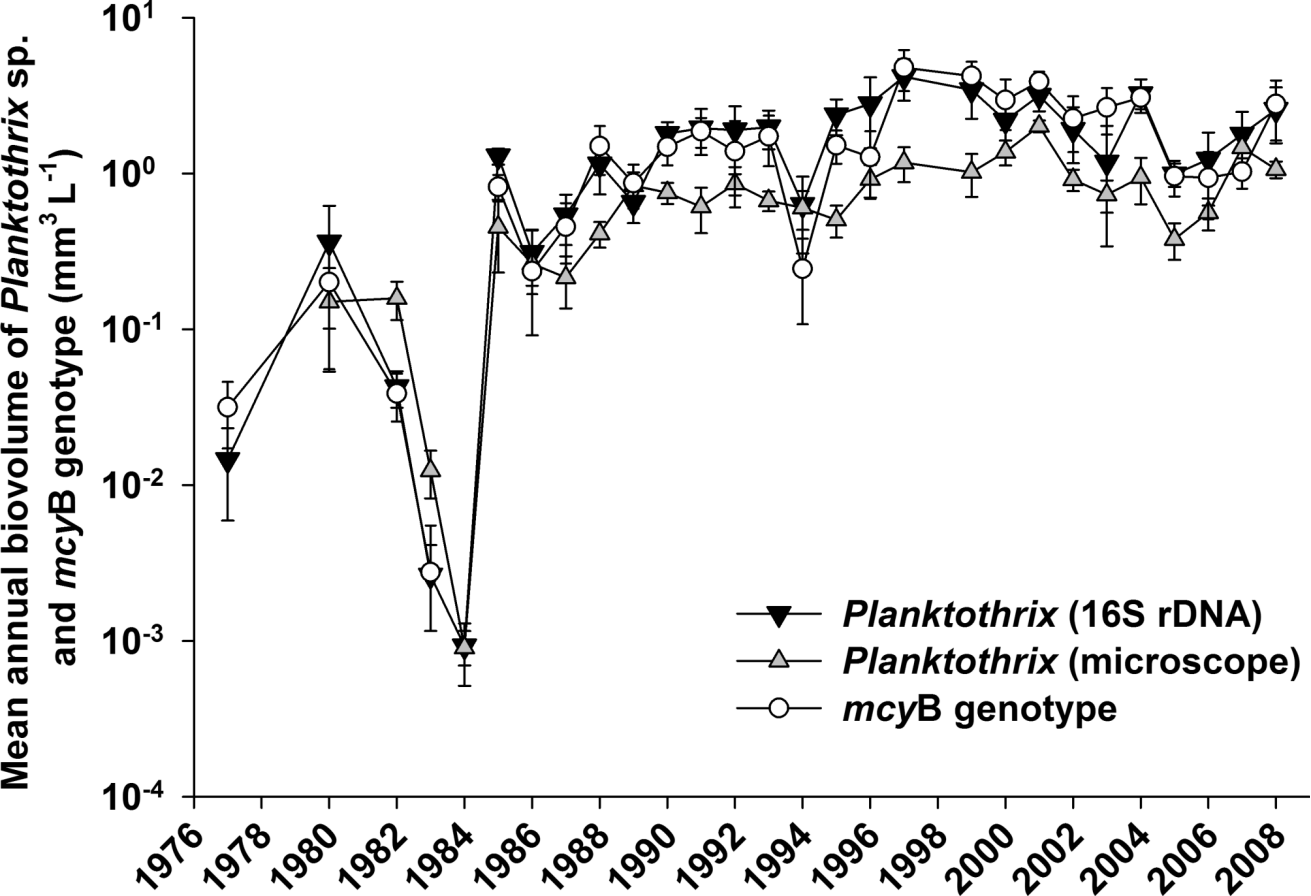
**Abundance of the total *Planktothrix *population and the toxic genotype**. Mean ± SE biovolume of *Planktothrix *determined in Lake Zürich from 1977 to 2008 using 16S rDNA and the *mcy*B gene by means of qPCR and microscopic counting. Biovolume in mm^3 ^L^-1^.

*Planktothrix *was detected by means of quantitative PCR (qPCR; using a Taq nuclease assay (TNA)) in all samples except for two (in the years 2002 and 2003). The mean *Planktothrix *biovolume per year was 1.6 ± 0.2 mm^3 ^L^-1^. The lowest annual *Planktothrix *biovolume was found in 1984 (0.001 mm^3 ^L^-1^) and the maximum annual biovolume was measured in 1997 (4.2 mm^3 ^L^-1^) (Figure 2). There was a highly significant positive and linear relationship between the *Planktothrix *biovolume quantified by 16S rDNA and the biovolume as determined by microscopic counting, *y *= -0.289 + 0.737*x*, *n *= 27, *R*^2 ^= 0.9, *P *< 0.001, where *x *is the average log_10 _biovolume (mm^3 ^L^-1^) per year as determined by 16S rDNA and *y *is the average log_10 _biovolume as determined by microscopic counting (Figure S1A in Additional file 1). The mean *Planktothrix *biovolume as calculated from microscopic counting was 0.71 ± 0.1 mm^3 ^L^-1^. The 16S rDNA abundance estimates were validated using a second TNA quantifying the phycocyanin intergenic spacer region (PC-IGS). Comparison of the biovolume as estimated by 16S rDNA and by the PC-IGS locus revealed a positive linear relationship following the equation *y *= -0.225 + 0.980*x*, *n *= 28, *R*^2 ^= 0.96, *P *< 0.001, where *x *is the average log_10 _biovolume (mm^3 ^L^-1^) per year determined by 16S rDNA and *y *is the respective log_10 _biovolume as determined via the PC-IGS (Figure S1B in Additional file 1). At four dates, the TNA for PC-IGS was negative, while the TNA for 16S rDNA was positive (three samples from 1984, and one sample from 1986). In all these cases the *Planktothrix *biovolume was very low (< 0.0006 mm^3 ^L^-1^), and the introduced variability was negligible. It is concluded that the TNA estimates on the *Planktothrix *abundance constitute a reliable estimate of the *Planktothrix *population density observed in the lake.

**Figure 3:**
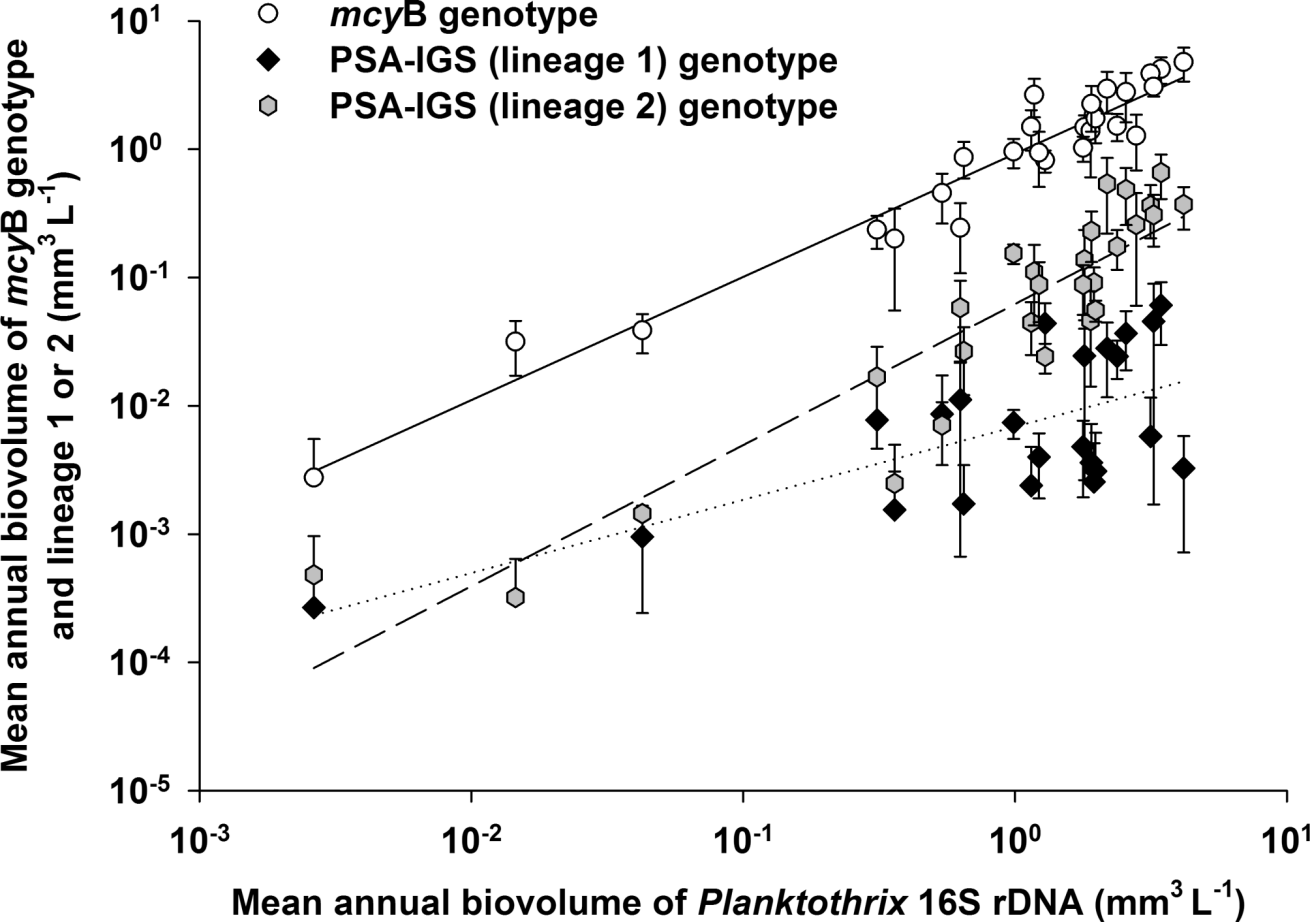
**Correlations of the biovolumes of the toxic genotype and two lineages differing in pigmentation with the total biovolume of *Planktothrix***. Relationship between the mean ± SE biovolume of *Planktothrix *as determined by 16S rDNA, the *mcy*-containing genotype and two clonal complexes of *Planktothrix*: lineage 1 (green-pigmented strains that lost the *mcy *gene cluster) and lineage 2 (red- or green-pigmented strains that always contain the *mcy *gene cluster). Biovolume in mm^3 ^L^-1^. Details on the regression curves are given in the text. PSA-IGS: P700 apoprotein subunit Ia and Ib intergenic spacer.

The TNA targeting the *mcy*B gene fragment indicative of the MC-producing genotype was found positive in 100 samples (87.8%). In 11 samples showing the lowest *Planktothrix *biovolume (< 0.0006 mm^3 ^L^-1^), no *mcy*B signal was recorded. A highly significant linear relationship was found between the abundance of the genotype carrying the *mcy*B gene fragment and the total population, *y *= -0.0358 + 0.960*x*, *n *= 27, *R*^2 ^= 0.94, *P *< 0.001 (Figure 3), where *x *is the average annual log_10 _biovolume (mm^3 ^L^-1^) as determined by 16S rDNA and *y *is the average annual log_10 _biovolume of the *mcy*B genotype. The mean proportion of the *mcy*B genotype was 106 ± 8% (minimum 38.9%, maximum 247.3%), indicating that the *Planktothrix *population was constantly dominated by the *mcy*B genotype (minimum 0.003 mm^3 ^L^-1^, maximum 4.8 mm^3 ^L^-1^, mean 1.6 ± 0.3 mm^3 ^L^-1^, Figure 2).

### Quantification of nontoxic *Planktothrix *in Lake Zürich

To elucidate whether the changes in environmental conditions affecting the phytoplankton composition also influenced the genotypic structure of the population, two types of nontoxic genotypes were quantified. First, genotypes containing the *mcy *gene cluster but inactive in MC production due to insertions by mobile elements or partial deletions of the *mcy *gene cluster [22]; and second, genotypes that lack the *mcy *gene cluster due to evolutionary gene loss [4].

**Figure 4:**
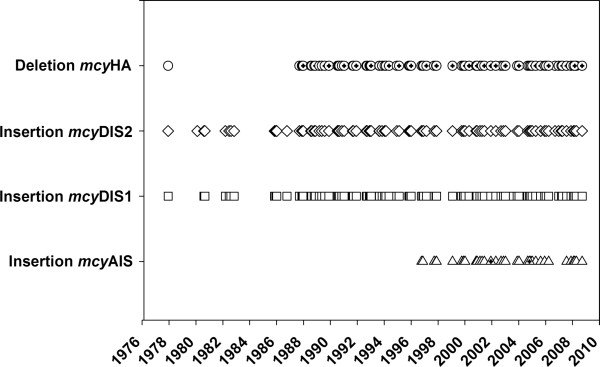
**Occurrence of inactive *mcy *genotypes between 1977 and 2008**. Occurrence of four different inactive *mcy *genotypes containing either insertions or a deletion within the *mcy *gene cluster. Genotypes were detected by means of qPCR. The cross in the symbols indicates numbers of the respective genotype that were above the quantification limit.

In general, all of the four genotypes containing either insertions or a deletion within the *mcy *gene cluster were detected frequently, but the majority in the lowest abundance (Figure 4). Only the genotype carrying a deletion between *mcy*H and *mcy*A occurred in sufficient amounts that could be quantified by the respective TNA. The *mcy*HA deletion was detected in 1977, although its numbers were below the quantification limit. It was not detected between 1980 and 1986, but occurred consistently later on: from 1987 to 2008, the genotype carrying the *mcy*HA deletion had an annual average proportion of 3.3 ± 0.4% (minimum 0.3%, maximum 7.2%) of the total population density as determined by 16S rDNA. In total, it was detected in 74% of the samples. Comparing its proportion between three decades (1977 to 1989, 1990 to 1999, 2000 to 2008) revealed no significant increase of this genotype (*P *= 0.16, Kruskal-Wallis one-way analysis of variance (ANOVA) on ranks). The other three inactive genotypes containing insertions were present in minimum concentrations only and could be detected in the undiluted DNA extract. The genotypes carrying insertions within *mcy*D, that is, *mcy*DIS1 and *mcy*DIS2, were detected in 87% and 88% of all the samples, respectively. These genotypes were detected throughout the investigated period except for the years 1983 and 1984, the years showing the lowest *Planktothrix *biovolume. By contrast, the *mcy*AIS genotype was detected as late as in 1996 and was then continuously present until 2008 (32% of the samples). It is concluded that inactive *mcy *genotypes occurred consistently, but never constituted a significant part of the total population.

Genotypes that lost the *mcy *gene cluster were quantified by amplifying a SNP in the *mcy*T gene, which constitutes a remnant of the *mcy *gene cluster in nontoxic strains [4]. In a first step, the sensitivity and efficiency of the TaqMan Genotyping Assay for *mcy*T was tested by the serial dilution of DNA isolated from axenic strains PCC7811 (allele 1, nontoxic genotype that lost the *mcy *gene cluster) and PCC7821 (allele 2, toxic genotype that still contains the *mcy *gene cluster) and analyzed using an allelic discrimination plot (Figure S2 in Additional file 1). The SNP assay showed a clear separation of the nontoxic versus toxic genotype and the two SNP probes were considered to be specific. The lowest DNA concentration that could be depicted was equivalent to two cells per template. Mixtures of the DNA (calculated from cell equivalents) from the two strains containing a 2% concentration of DNA from nontoxic strain PCC7811 showed a slight deviation of the delta Rn values compared with those of pure DNA from the toxic strain PCC7821. The delta Rn values of DNA mixtures with concentrations of 10% and 20% of nontoxic strain PCC7811 were clearly different from pure DNA of the toxic strain PCC7821, while the concentration of 50% of DNA of the nontoxic strain PCC7811 was displayed in the center of the allelic discrimination plot (Figure S2 in Additional file 1). It is concluded that the nontoxic genotype was detected as soon as its share exceeded 2% of the total population.

**Figure 5:**
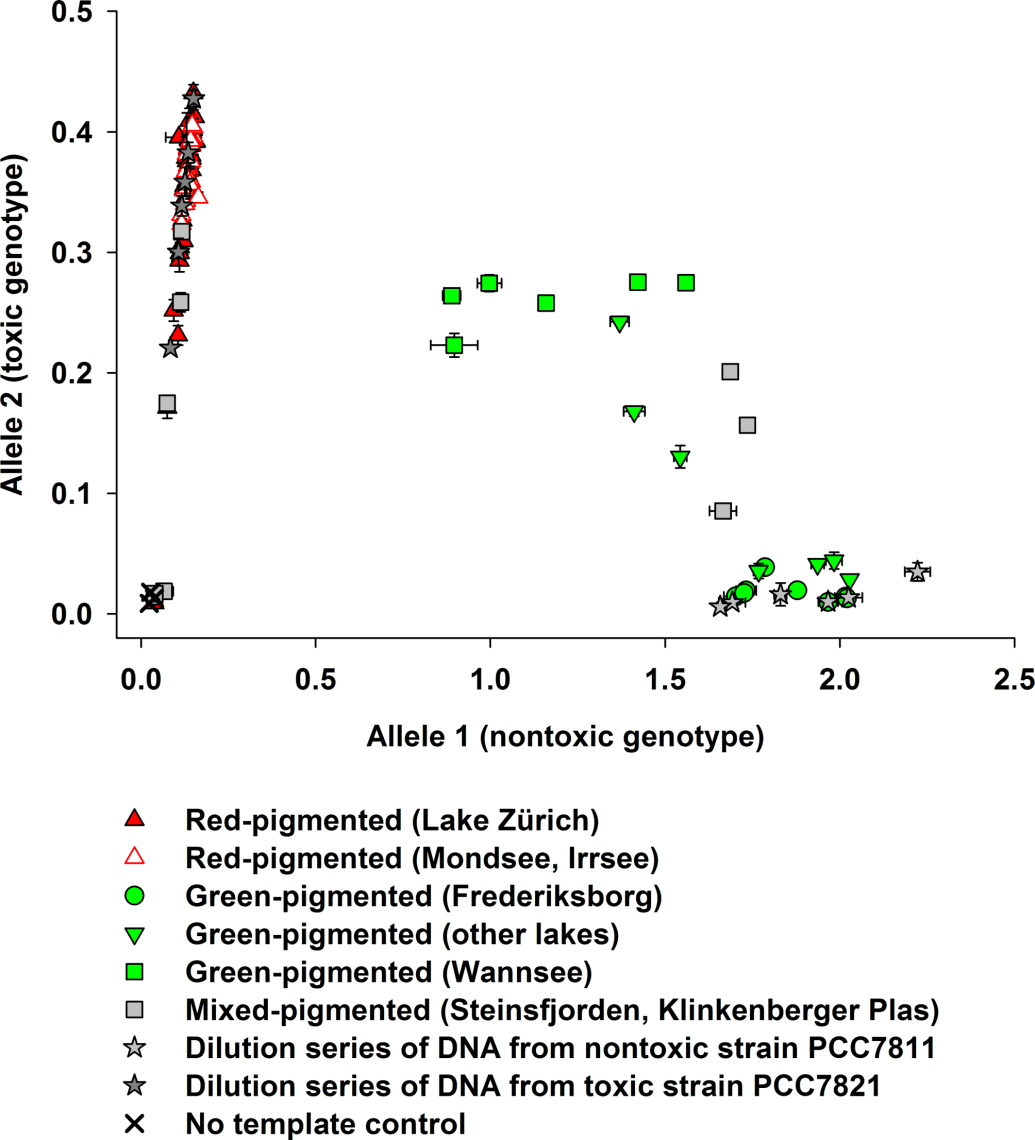
**Composition of the toxic and nontoxic genotype in populations of *Planktothrix *across Europe**. Allelic discrimination plot of toxic and nontoxic *Planktothrix *genotypes of populations differing in pigmentation as determined by a custom TaqMan SNP genotyping assay within *mcy*T, indicative of a genotype that lost (allele 1) or still contains (allele 2) the *mcy *gene cluster. Delta Rn values represent the difference in fluorescence from the post-PCR and pre-PCR reads of the normalized reporter (VIC, FAM). The symbols represent the mean ± SE of measurements in triplicate. When not visible, the error bars are hidden behind the symbol. Values located close to the no-template control failed to amplify.

The analysis of field samples obtained from Lake Zürich and other shallow eutrophic lakes (Table 1) by an allelic discrimination plot revealed four distinct clusters (Figure 5). The first cluster comprised samples clustering along the *y*-axis (allele 2, toxic genotype), indicating populations only composed of the toxic genotype. This cluster included samples dominated by the red-pigmented ecotype: Lake Zürich, Irrsee and Mondsee and one lake with a population of the red- and the green-pigmented ecotype (Steinsfjorden). Samples of the green-pigmented populations (Wannsee, Slotermeer, Tjeukemeer and Havel) formed a second cluster in the center of the allelic discrimination plot, indicating populations comprising both the toxic and the nontoxic genotype. The *Planktothrix *population tested from Klinkenberger Plas comprising the red- and the green-pigmented ecotype was also found in this central cluster. By contrast, samples from shallow polymictic lakes (for example, Lake Frederiksborg Slotssø, Zeegerplas and the Albufera Lagoon) with a green-pigmented *Planktothrix *population formed a third cluster along the *x*-axis (allele 1, nontoxic genotype), indicating populations composed entirely of the nontoxic genotype. It is concluded that, in Lake Zürich, the nontoxic genotype carrying the *mcy*T gene as a remnant of the *mcy *gene cluster never became abundant during the entire observation period. By contrast, *Planktothrix *populations that were either only green-pigmented or both red- and green-pigmented typically showed a higher proportion of the nontoxic genotype.

**Table 1:** Origin of field samples analyzed by the TaqMan Genotyping Assay (SNP) for allelic discrimination of the *mcy*T gene both as a remainder of the *mcy *gene cluster (nontoxic strains) and as part of the *mcy *gene cluster (toxic strains) [4,12].

Lake	Country	Number of samples	Lake area (km^2^)	Z_mean_ (m)	Z_max_ (m)	Sampling period
**Red-pigmented populations^a^**						
Lake Zürich	CH	51	68	52	136	1977-2008
Irrsee	AT	4	3.5	15	32	Sep-Dec 2003
Mondsee	AT	22	16	36	68	Apr 2003-Dec 2004
**Green-pigmented populations**						
Albufera Lagoon	ES	1	21	1	3	Aug 2004
Frederiksborg Slotssø	DK	8	0.2	3	9	Jul-Oct 2003
Havel (Potsdam)	DE	1	-	3	4	Sep 2004
Slotermeer	NL	1	12.4	1.2	6	May 2004
Tjeukemeer	NL	1	20	2	5	Oct 2004
Zeegerplas	NL	3	0.7	18	34	Aug, Sep and Oct 2004
Wannsee	DE	6	2.7	6	9	Feb-Mar and May-Aug 2000
**Mixed-pigmented populations**						
Klinkenberger Plas	NL	3	0.3	-	30	May, Jul and Aug 2004
Steinsfjorden	NO	6	13.9	10	24	Jul-Sep 2003, Jul 2004

^a^Pigmentation type was determined by visual inspection in the microscope. AT: Austria; CH: Switzerland; DE: Germany; DK: Denmark; ES: Spain; NL: Netherlands; NO: Norway.

### Quantification of the pigmentation types of *Planktothrix *in Lake Zürich

To find out whether the abundance of the green-pigmented (nontoxic) ecotype was influenced by the trophic change during the study period, two phylogenetic lineages differing in pigmentation and the presence of the *mcy *gene cluster were quantified using the P700 apoprotein subunit Ia (*psa*A) and Ib (*psa*B) intergenic spacer region (PSA): lineage 1, the green-pigmented, nontoxic genotype that lost the *mcy *gene cluster (PSA I), and lineage 2, both red- and green-pigmented toxic genotype that always carried the *mcy *gene cluster (PSA II) [4]. The green-pigmented lineage 1 was detected during the entire observation period although in a smaller number of samples (43%). By contrast, lineage 2 (red- and green-pigmented ecotypes containing the *mcy *genes) was detected in 83% of all the samples. A few samples with low *Planktothrix *biovolume (< 0.09 mm^3 ^L^-1^) did not show a TNA signal indicative of lineage 2 (17% of the samples). The average proportion of the genotype of lineage 1 was generally low (minimum 0%, maximum 3.8%, mean 0.9 ± 0.2%) and no increase or decrease was observed when comparing the mean proportion of this genotype between the three decades: 1977 to 1989: 1.3 ± 0.5% (*n *= 9), 1990 to 1999: 0.6 ± 0.2% (*n *= 9) and 2000 to 2008: 0.7 ± 0.2% (*n *= 9); *P *= 0.26. Maximum abundances were measured in 1983 and 1985 (3.8% and 3.5% of the total population), coinciding with the time of the *Planktothrix *population breakdown and the following recovery. The mean proportion of the genotype of lineage 2 was 7.3 ± 1% of the total population (minimum 0.2%, maximum 18.5%). A significant increase of this genotype was detected when comparing the mean proportion between the three decades: 1977 to 1989: 2.3 ± 0.5% (*n *= 9), 1990 to 1999: 7.5 ± 1.5 (*n *= 9) and 2000 to 2008: 12.1 ± 1.2% (*n *= 9); *P *< 0.001.

The abundances of genotypes of lineage 1 and 2 were both linearly correlated to the total population as follows: lineage 1, *y *= -2.165 + 0.57*x*, *n *= 23, *R*^2 ^= 0.45, *P *< 0.001 (dotted line, Figure 3); lineage 2, *y *= -1.207 + 1.1*x*, *n *= 27, *R*^2 ^= 0.83, *P *< 0.001, (dashed line, Figure 3), where *x *is the log_10 _biovolume of the total population determined by 16S rDNA (mm^3 ^L^-1^) and *y *is the log_10 _biovolume of *Planktothrix *lineage 1 or 2 (mm^3 ^L^-1^), respectively. The regression curve of lineage 1 significantly differed in intercept and slope from the regression curve obtained between the *mcy*B genotype and the total population (*P *< 0.001, *n *= 27). By contrast, the regression curve of lineage 2 significantly differed in intercept but not in the slope (*P *= 0.179, *n *= 27) from the *mcy*B regression curve. Thus, where lineage 2 showed a parallel increase with the abundance of the total population, lineage 1 showed a tendency to decrease in proportion with a higher *Planktothrix *population density. The same results were obtained when the samples from the years with lowest *Planktothrix *density (1977 and 1982 to 1984) were excluded (data not shown). It is concluded that the genotype of lineage 2 consistently outgrew the genotype of lineage 1 under conditions of favorable growth for the *Planktothrix *population.

## Discussion

### Dominance of red-pigmented *Planktothrix *in Lake Zürich

The observed increase of the *Planktothrix *population in Lake Zürich over the last 30 years can be explained by the improved light regime in the water column that occurred following re-oligotrophication, through the disappearance of eukaryotic algae blooms. This phenomenon has already been documented for other lakes [2]. It is well known that *P. rubescens *stratifies in the metalimnion of thermally stratified lakes at approximately 12 m depth, and grows at the lowest irradiance [23]. Furthermore, *Planktothrix *can take advantage of higher concentrations of the limiting macronutrient (soluble reactive phosphorus) in the metalimnion as well as intracellularly stored phosphate for three or four further cell divisions [1,24]. The population breakdown in 1984 was probably the consequence of various factors that weakened the population. First, higher biomasses of eukaryotic algae occurred during the years 1983 and 1984 and might have reduced deep light penetration and forced *Planktothrix *to enter the mixed epilimnion [25]. Mur [26] reported a higher growth rate of the green algae (*Scenedesmus*) at high light intensities whereas *Planktothrix *grew more efficiently at the lowest light intensities, thereby outcompeting *Scenedesmus*. Second, Posch *et al. *[27] reported that holomixis occurred in Lake Zürich from 1980 to 1987, probably leading to major losses in the *P. rubescens *population. Walsby and co-workers [17] reported a major decrease in population biomass in spring after a strong winter 1996 to 1997) when compared with population biomass in spring after a warmer winter (1994 to 1995). Posch *et al. *[27] further suggested that the increased epilimnetic water temperatures since 1988 led to a more stabilized stratification of the water column and allowed the survival of a bigger share of the population over winter due to reduced loss processes during lake mixing. However, it is unclear whether changes in environmental parameters (for example, depth of the euphotic zone or higher stability of the water column due to rising water temperatures) may not only have an impact on the growth of the total population but also on the subpopulation dynamics of specific ecotypes.

### Factors indirectly causing the stability of genotype composition

Strikingly, the genotypic composition of *Planktothrix *was found to be rather stable over the last 30 years. The population was constantly dominated by the toxic *mcy*-containing genotype, while the share of the nontoxic (*mcy-*lacking) genotype was low. Sabart *et al. *[28] found the proportions of toxic and nontoxic genotypes in the populations of *Microcystis *along the catchment of River Loire to be generally stable over time but variable between sampling sites. Okello *et al. *[29] reported differing proportions of toxic and nontoxic genotypes from spatially isolated *Microcystis *populations in Uganda, and these differences in proportions were much more pronounced than the observed seasonal variation within lakes. These findings are in accordance with the present study and imply the role of both abiotic and biotic parameters of the habitat (for example, light regime, lake morphometry, zooplankton grazers, viruses) that regulate genotype composition [12]. It is important to note that these factors are not directly related to MC production and that effects of re-oligotrophication on the *Microcystis *genotype structure will differ considerably from those on the *Planktothrix *genotypic composition because of the overall difference between the ecological niches of the two taxa [14].

Probably the best example for an ecological trait not directly related to MC production is green versus red pigmentation. In Lake Zürich, the green-pigmented nontoxic *Planktothrix *lineage 1 was detected throughout the study period, but in very low proportions only. Interestingly, in the period before and after the population breakdown, a maximum proportion of the green-pigmented lineage 1 was observed. During this eutrophic period, blooms formed by non-stratifying phytoplankton still occurred (Figure 1). Under these conditions, the green-pigmented ecotype might have been at a selective advantage because of the variable irradiances, as it is found to be characteristic of shallow polymictic lakes [14]. By contrast, the red-pigmented ecotype was shown to be a better competitor for light only under mesotrophic and stratified conditions as its compensation depth for growth was lower compared with that of the green-pigmented ecotype [13]. Consequently, under eutrophic strong light shading conditions, the red-pigmented *Planktothrix *are seldom abundant [14,30].

Because Lake Zürich is a deep lake, the resistance of gas vesicles to hydrostatic pressure may be a critical factor for survival during conditions of lake mixing, as gas vesicles may collapse and filaments cannot return to the euphotic zone. It has previously been shown that the red- and green-pigmented ecotypes of *Planktothrix *vary in the presence of gas vesicle proteins, which could be attributed to lake depths [18]. The filaments remaining buoyant after mixing will, therefore, form the basis of the population for the next season [17]. Gas vesicles resisting strong hydrostatic pressures are more commonly found among the red-pigmented strains of *Planktothrix*, as in the population of Lake Zürich, whereas gas vesicles that collapse at a relatively low critical pressure are more common within green-pigmented populations [18]. Additionally, the genotype that produces gas vesicles that resist the highest hydrostatic pressures, which is the dominant genotype in Lake Zürich, was not detected in isolates obtained from 21 Norwegian lakes with a maximum depth of 67 m [18]. Accordingly, in red-pigmented populations, stronger gas vesicle types were found to be positively related to lake depth [31].

It is likely that the green-pigmented *Planktothrix *ecotype of Lake Zürich produces gas vesicles that do not resist the critical pressure during lake mixing and that only a small percentage of filaments survive entrainment in the hypolimnion. Therefore, in spring, the basis for the population development of the green-pigmented ecotype would be low when compared with the red-pigmented ecotype, of which a larger proportion can survive mixing during the winter (> 90% of the filaments [17]). Our own preliminary results showed that strains of nontoxic lineage 1 almost exclusively contained the weakest gas vesicle genotype (gvpC^28^) while strains of toxic lineage 2 typically contained two gas vesicle genotypes (gvpC^28 ^and gvpC^20^). Some of the strains of lineage 2 also showed the genotype encoding the smallest gas vesicle (gvpC^16^), which is known to resist high hydrostatic pressure [18].

### Potential factors directly favoring microcystin production

The inactive genotypes formed a stable but very small subpopulation throughout the study period. By contrast, the genotype carrying an insertion within *mcy*A (*mcy*AIS) was detected for the first time in 1996. It is conceivable that this genotype arrived in the lake at a later time than the other inactive genotypes, given that all of these four inactive genotypes occurred regularly in different populations in the Alps [22]. It is concluded that these inactive *mcy*-containing genotypes grew very slowly and still unknown factors keep their abundance low.

The role of MC as a feeding deterrent has been investigated and evidence has been gathered that MCs are indeed toxins resulting in a significantly reduced survival rate of herbivorous crustaceans [7,32,33]. The frequently cited hypothesis that MC-producing cyanobacteria evolved before their potential predators (the metazoans), which is based on phylogenetic analyses [34], ignores the fact that chemical defense still could be an additional (secondary) function simply because MCs are very effective toxins (for example, see [35]). In addition, the evolutionary age of eukaryotes has been a matter of debate, and the coexistence of eukaryotes with prokaryotes has been inferred from biomolecules in fossil oil droplets with an age of 2.4 billion years [36]. Thus, the possible co-occurrence of cyanobacteria and eukaryotes before snowball earth (2 billion years ago) is a matter of ongoing research. We suggest that, in nontoxic *P. agardhii*, other bioactive but nontoxic peptides functionally replace MC. For example, both lysogenic bacteria [37] and parasitic fungi have been described; for example, Sønstebø and Rohrlack [38] reported a relationship between chytridiomycete infectability of strains and the presence of certain peptides, such as cyanopeptolins and anabaenopeptins. The higher variability in toxic and bioactive peptide genotype proportion among nontoxic green-pigmented populations could be because of an ongoing co-evolutionary arms race between host and parasite [38].

As suggested for *Microcystis *[39], MC is considered to have an intracellular impact on the stability and activity of proteins involved in carbon-nitrogen metabolism by interfering with their redox state control. The enhanced binding of MC to proteins is thought to be part of a general response to oxidative stress [39,40]. The binding of MC to cysteine residues of proteins might be stimulated by conformational changes, which delays the degradation of redox-sensitive proteins. Zilliges *et al. *[39] showed that a MC-deficient mutant of *Microcystis *was more susceptible to high irradiance compared with the MC-producing wild type. It is conceivable that, in particular for red-pigmented *Planktothrix*, conditions of enhanced oxidative stress occur, for example when the low-light-adapted buoyant *Planktothrix *population accumulates at the surface in the autumn [41]. It is known that, among all phytoplankton species, red-pigmented *Planktothrix *is most efficient in light harvesting but this capability increases the chance of light damage to the cells under high irradiance, such as during calm days at the water surface [41]. Green-pigmented *P. agardhii *has been found more resistant to high light intensities [15]. In light of these findings, it can be speculated that MC production would be of selective advantage in red-pigmented *Planktothrix *populations, whereas MC production might be of less selective value in green-pigmented populations due to the general lower sensitivity to high light damage. Further, *mcy *genotypes inactive in MC production in red-pigmented populations would be selectively reduced during surface blooms under high irradiance conditions. It is possible that *mcy *genotypes inactive in MC synthesis grow under physically stable stratified conditions of the water column, as losses due to accidental accumulation at the surface will be low.

## Methodology

Field studies on toxic genotype composition within a population often cover one season only and rarely exceed a period of several years. Long-term studies on the genotypic composition of a population with regard to environmental parameters would, therefore, be of relevance to understand the influence of lake restoration measures on bloom toxicity. A longer study period can also aid in predicting the toxic genotype composition during future blooms with regard to changing environmental conditions. Historical analyses investigated the occurrence of cyanobacteria by extracting DNA or MC from dried biomass archived in herbaria or from water samples and sediment cores [42-44]. These samples offer a reservoir of precious information about preserved plankton organisms. A set of continuously taken and preserved samples spanning decades is, however, scarce.

It is known that analyzing DNA from ancient samples causes difficulties either because of DNA degradation or contamination by contemporary DNA. Ancient DNA is often highly fragmented (100 to 500 bp) and contains modifications that hinder the amplification by Taq polymerases. Rapid desiccation can, however, delay enzymatic or microbial degradation processes [45]. Schober and Kurmayer [46] investigated the influence of freeze-drying on the quantification of cells by qPCR. When compared with cell numbers estimated from aliquots stored at -20°C, no differences were detected between the two treatments. We chose qPCR for estimating the proportions of genotypes from preserved samples for a number of reasons: fragmentation of DNA is considered of minor importance as the size of the amplification products is generally small (< 100 bp); estimating proportions of individual genotypes (relative to the total population) should be robust against the bias due to point mutations in primer-binding regions; and the absolute cell concentrations as determined by qPCR could be validated by the cell numbers as determined in the microscope. On an absolute scale, a rather high correlation was observed between the biovolume of the total population estimated by microscopic counting and as estimated by 16S rDNA or the PC-IGS (Figure S1 in Additional file 1). The qPCR approach to relate the subpopulation of the toxic genotype to the total population was developed a decade ago [47], and typically the subpopulation of the nontoxic genotype has been inferred only indirectly. In this study, the first attempt was made to quantify the subpopulation of the nontoxic genotype directly by using a SNP that is indicative of the nontoxic genotype that lost the *mcy *gene cluster (Table S1 in Additional file 1[4]). In fact, the lowest proportion of the nontoxic genotype in all the samples from Lake Zürich as revealed by the allelic discrimination plot confirms that the population was constantly dominated by the toxic genotype (Figures 2 and 5). In addition, the allelic discrimination plot results highly correlate with the proportions of the *mcy*B gene as estimated in other red- and green-pigmented populations [12].

For unknown reasons, the sum of the PSA I and PSA II genotypes did not make up 100% of the population, but on average constituted only 8.2 ± 1.7%. It is speculated that the relatively larger amplicon size of both the PSA I and PSA II TNAs (167 bp and 158 bp) could be responsible for the relative underestimation of those genotypes. The fact that isolated DNA from ancient tissues is generally fragmented has already been described [48,49]. In our study, the isolated DNA of samples from different years was inspected on an agarose gel and showed a high degree of fragmentation. We therefore conclude that the underestimation of both PSA-IGS genotypes can be attributed to the relatively long amplification size that, except for the *mcy*AIS TNA, exceeds the size of all other TNAs (Table 2). However, since TNAs for both PSA-IGS genotypes were designed from the same primer-binding region of the same locus and the calibration curves could not be discriminated in slope or in intercept, the proportions of both PSA-IGS genotypes are considered reliable.

**Table 2:** Calibration curves and strains used for the Taq nuclease assay and the TaqMan genotyping assay (SNP).

TNA	Gene locus	Strain	Calibration curve^a^	*E *(%)^b^	*R*^2 c^	n	Quantification limit (cells per template)^d^	Amplicon length (bp)
16S	16S rDNA	PCC7821	*y *= 11.021 - 3.5123*x*	92.6	0.997	12	4	82
PC-IGS	Intergenic spacer of the phycocyanin operon	PCC7821	*y *= 9.9401 - 3.5673*x*	90.7	0.994	12	4	72
*mcy*B	First adenylation domain of *mcy*B	PCC7821	*y *= 12.151 - 3.3745*x*	97.9	0.993	11	4	76
*mcy*DIS1	3'-end of IS-element within *mcy*D	No.110	*y *= 7.941 - 3.8823*x*	81.0	0.994	12	4	93
*mcy*DIS2	3'-end of IS-element within *mcy*D	No.139	*y *= 11.956 - 3.1314*x*	108.6	0.980	11	18	110
*mcy*AIS	3'-end of IS-element within *mcy*A	No.40	*y *= 11.126 - 3.6644*x*	87.5	0.991	11	4	168
*mcy*HA	Deletion between *mcy*H and *mcy*A	No.62	*y *= 14.648 - 3.751*x*	84.8	0.996	12	4	71
PSA I (lineage 1)^e^	Intergenic spacer between *psa*A and *psa*B	PCC7811	*y *= 14.144 - 3.4482*x*	93.2	0.996	11	3	167
PSA II (lineage 2)^e^	Intergenic spacer between *psa*A and *psa*B	PCC7821	*y *= 13.681 - 3.3161*x*	100.2	0.978	9	4	158
SNP Assay allele 1	SNP within *mcy*T (genotypes that lost the *mcy *gene cluster)	PCC7811	*y = *10.449 - 3.6685*x*	98.2	0.996	15	2	66
SNP Assay allele 2	SNP within *mcy*T (genotypes containing the *mcy *gene cluster)	PCC7821	*y = *15.875 - 3.3579*x*	98.5	0.996	15	2	66

^a ^Calibration curve = regression line; *y *= number of PCR cycles at the threshold fluorescent value (C_t_), *x *= amount of template DNA (expressed as log_10 _of mm^3 ^biovolume per template). ^b ^Amplification efficiencies (*E*) were calculated from *E *= (10 ^-1/*x *^-1) × 100 (%), *x *= slope of calibration curve. ^c ^*R*^2 ^= coefficient of determination of calibration curve. ^d ^Quantification limit given as cell number equivalents represents the lower end of the calibration curve. ^e ^According to [4].

## Conclusions

The stability in genotype composition provides evidence for the competitive superiority of a phylogenetic lineage that was shown to retain the MC gene cluster during evolution [4]. From an evolutionary point of view, it is important to see that the evolution of toxin synthesis genes within this population of cyanobacteria is slow. The long-term stability of toxic genotype composition, however, is useful to forecast the toxicity of blooms formed by *Planktothrix *in deep mesotrophic water bodies. In future, it will be necessary to consider the evolutionary history of the toxic genotypes of cyanobacteria to unravel their selective adaptations to the various aquatic environments.

## Methods

### Study site

Lake Zürich in Switzerland (47°15'N, 8°38'E, 406 m above sea level) is a deep (mean depth 51 m, maximum depth 136 m), monomictic lake with a surface area of 68 km^2^. The first signs of Lake Zürich eutrophication were observed before 1890 (problems with the clogging of drinking water filters due to high zooplankton biovolumes). At the peak of eutrophication in 1965, the total phosphorus concentration reached a maximum of 100 μg L^-1 ^at spring overturn [50] and the *Planktothrix *abundance was close to zero from 1965 to 1975. Since 1975, the *Planktothrix *population has recovered and has an average share of 33% per year (minimum 0.1%, maximum 68%) of the total phytoplankton biovolume between 1980 and 2008. Today, Lake Zürich is classified as mesotrophic with average total phosphorus concentrations of 23 μg L^-1 ^(minimum 3.9 μg L^-1^, maximum 78.1 μg L^-1^) between 1980 and 2008. For the analysis of both phytoplankton and total phosphorus concentrations (Figures 1 and 2 and Figure S1A in Additional file 1), the average of the countings between the surface and 20 m in depth (0, 1, 2.5, 5, 7.5, 10, 12.5, 15 and 20 m) of the monthly sampling data was used.

### Extraction of DNA from archived phytoplankton samples

A total of 111 samples from 1977 to 2008 were analyzed (in 1981 and 1998, no sampling was performed). The samples originated from a biweekly monitoring program targeting the euphotic zone by separate filtration of two liters from depths of 0, 5, 10 and 20 m through glass fiber filters, which were subsequently dried at 110°C for 2 h and stored in a desiccator until the next day. The filters were glued on paper and stored at room temperature (Figure S3 in Additional file 1). Filters were inspected and four filters from each year that showed maximum biomass were used for DNA extraction. Thus, the sampling depth varied according to the occurrence of the maximum population density. From the majority of the samples, one half of the filter with the preserved phytoplankton was used for DNA extraction, from ten samples only one quarter of the filter was used, and from three samples (600 mL from different depths, 7 December 1977) the phytoplankton of the complete filter was used for DNA extraction.

DNA was extracted from filters using the chloroform-phenol method as previously described [51]. The extracted DNA was precipitated from the aqueous phase by adding half the sample volume of ammonium acetate (7.5 M), one tenth of the sample volume of magnesium chloride (0.1 M), 1 μL of glycogen (Fermentas, St. Leon-Rot, Germany) and one sample volume of isopropanol before overnight incubation (-20°C). After centrifugation (16,000 g, 4°C, 1 h) the supernatant was discarded and samples were washed twice with 70% ethanol by centrifugation (20 min, 4°C). The DNA pellets were dried in a sterile hood and resuspended in 50 μL of sterile Millipore water.

### Design of qPCR TaqMan assays (Taq nuclease assay)

Samples were analyzed by TNA for the total population of *Planktothrix *by the 16S rDNA locus [11] and the PC-IGS [46]; for the abundance of the *mcy*B genotype encoding MC synthesis [12]; for the abundance of various mutant *mcy *genotypes carrying either a deletion or insertion inactivating MC biosynthesis [22], such as *mcy*DIS1, *mcy*DIS2, *mcy*AIS (insertion of a transposable element into the *mcy *gene cluster) and *mcy*HA (deletion of 1.8 kbp between *mcy*H and *mcy*A); for the abundance of the nontoxic genotype that lost the *mcy *gene cluster [4]; and for the abundance of red-pigmented versus green-pigmented ecotypes via the IGS between *psa*A and *psa*B (PSA-IGS; see below). The nontoxic genotype that lost the *mcy *gene cluster, except for *mcy*T, was differentiated from the genotype still containing the *mcy *gene cluster using a SNP. The primers and TaqMan probes for the 16S, PC-IGS, the *mcy*B gene and the four inactive *mcy *genotypes have been published previously (Table 2). For the design of TNAs targeting the PSA-IGS locus, sequences from 62 *Planktothrix *strains (GenBank:EU258202 to EU258263[4]) were aligned together with 78 sequences of *Planktothrix *strains isolated from Europe, North America and East Africa (R. Kurmayer, unpublished) by means of Clustal W 2.0. A target region was identified that discriminates all the strains of lineage 1 (*n *= 56; containing strains that are green-pigmented and lost the *mcy *gene cluster) from strains of lineage 2 (*n *= 84; containing both red-pigmented and green-pigmented strains that always retained the full *mcy *gene cluster). The following forward primers, TaqMan probes and reverse primers were designed: lineage 1 (167 bp): forward primer PSA I + (5'-CCAGCAATTCAACCTCGC-3'), TaqMan probe PSA I (5'-TGGTGTAGCTCACTACCTCTTAGGAGGCAT-3') and reverse primer PSA I- (5'-AAAGTAGATTAGATTTCCTCCCACCT-3'); lineage 2 (158 bp): forward primer PSA II + (5'-CCAGCAATTCAACCTCGC-3'), TaqMan probe PSA II (5'-CGTGCGGTTGGTGTAGCTCACTACC-3') and reverse primer PSA II- (5'-ATTCAGCCTTTCCCAGTCCC-3'). Both probes were labeled with 6-carboxyfluorescein (FAM) at the 5'-end and BlackBerry Non-Fluorescent Quencher (NFQ) at the 3'-end.

### Detection of the nontoxic genotype by single nucleotide polymorphism (custom TaqMan genotype assay)

In total, 113 *mcy*T sequences (GenBank: EU266304 to EU266364, and RK, unpublished; Table S1 in Additional file 1) of both toxic and nontoxic strains were aligned. A SNP was found to discriminate all the strains that lost the *mcy *gene cluster from strains that still contain the full *mcy *gene cluster. A custom TaqMan SNP genotyping assay based on this SNP was created using the Custom TaqMan Assay Design Tool (Applied Biosystems, ABI, Vienna, Austria) to differentiate the two genotypes. The following forward and reverse primers and two TaqMan probes labeled with 4,7,2'-trichloro-7'-phenyl-6-carboxyfluorescein (VIC) or FAM at the 5'-end and a NFQ at the 3'-end were obtained: *mcy*T SNP + (5'-ACAGAGAAAGCCGAGTTGGTT-3'), *mcy*T SNP-(5'-AGATTTGAAACCTAACGCCTTGGA-3'), TaqMan Probe allele 1 (VIC 5'- TGTTCCCACCAAGCTT-3' NFQ) and TaqMan Probe allele 2 (FAM 5'- CCCGCCAAGCTT-3' NFQ) (66 bp). The specificity and sensitivity of the assay were tested by measuring the mixtures of DNA from axenic strains PCC7811 (loss of the *mcy *gene cluster except the *mcy*T gene) and PCC7821 (containing the *mcy *gene cluster).

### Setup of qPCR TaqMan assays

All the samples were measured in triplicate in a total volume of 25 μL including 5 μL of template DNA. The initial denaturation of 10 min at 95°C was followed by 50 cycles of a two-step PCR with an annealing and elongation temperature of 60°C or 55°C on an Eppendorf Master Cycler Ep Realplex system (Eppendorf, Vienna, Austria), as described [11]. To increase the DNA concentration in the template as much as possible, the inactive *mcy *genotypes were measured in a total volume of 12.5 μL including 1 μL of the pure DNA extract and 1 μL (50 μg μL^-1^) of bovine serum albumin. Because these results were frequently below the limit of quantification, only a presence/absence analysis was performed.

TaqMan assays were tested for specificity and sensitivity using dilution series of DNA isolated from *Planktothrix *strains 110, 139, 40 and 62, as described (Table 2) [22]. Calibration curves were also prepared from a dilution series of DNA from strains PCC7811 and PCC7821 for the TNA PSA I and PSA II, representing lineage 1 or lineage 2 [4]. Strains PCC7811 and PCC7821 were grown at 20°C in a BG11 medium under continuous light (5 to 15 μmol photons m^-2 ^s^-1^) and the cells were harvested during logarithmic growth. For each TNA calibration curve, the predetermined DNA concentrations in the template (expressed as biovolume per template) were diluted and related to the measured C_t _values [22]. The limit of quantification was defined as the lowest concentration of the calibration curve, which corresponded to 3 to 18 cells per template for the individual assays (Table 2). In the case that the C_t _values of a particular gene locus were below the limit of quantification, only the results from the 16S rDNA locus were included in the analysis (*n *= 8).

### TaqMan Genotype Assay (single nucleotide polymorphism)

All the samples were measured in triplicate on a ViiA7 Real-Time PCR System (ABI, Vienna, Austria), using the ViiA7 software (version 1.2.1) in a total volume of 12.5 μL including 2.5 μL of template DNA, ABI TaqMan Universal PCR Master Mix, the SNP Genotyping Assay Mix and sterile water according to the manufacturer's recommendations. A two-step PCR of 55 cycles was run with denaturation (92°C, 15 s) and annealing and extension (60°C, 1 min). The protocol included an initial denaturation step at 92°C for 10 min and a pre-read and post-read step at 60°C for 30 s, each. The relative threshold cycle was automatically set to 0.04 for all reads. The results were analyzed based on the delta Rn values (with Rn = fluorescence signal of reporter dye normalized to the fluorescence signal of the passive reference ROX) for allele 1 and allele 2, which represent the difference of the normalized fluorescence signal (VIC, FAM) between the pre-PCR and the post-PCR read.

To test the sensitivity and the specificity of the SNP assay, DNA of the axenic nontoxic strain PCC7811 was mixed down to 0.2, 0.5, 1, 2, 10, 20 and 50% of DNA from the toxic strain PCC7821 per template (calculated as cell equivalents). Dilution series from DNA of axenic strains PCC7811 and PCC7821 reaching from 2 to 180,000 and 2 to 230,000 cells per template, respectively, were established. At least one sample per year (1977 to 2008, *n *= 35), as well as depth integrated and net samples (0 to 20 m) of Lake Zürich from the years 2005, 2006 and 2007 (*n *= 16 [11]) were analyzed. In addition, samples of various European lakes composed of either green- or mixed-pigmented *Planktothrix *populations [12] were included (Table 1).

### Statistical analyses

To calculate regression curves, the raw data were log_10_-transformed and tested for normal distribution (Shapiro-Wilks, *P *> 0.05) and for constant variance by computing Spearman's rank correlation between the absolute values of the residuals and the observed value of the dependent variable (*P *> 0.05). The residuals were tested for their independence from each other by the Durbin-Watson statistic.

The linear regressions between the total population density (as estimated from 16S rDNA) and the abundance of the *mcy*B gene and the two PSA-IGS genotypes (representing lineage 1 and lineage 2) were compared in terms of the slope and intercept using a general factorial model of ANOVA [52]. A SPSS statistical package (V 19.0 for Windows) was used. To compare the genotype proportions from samples between the three decades (1977 to 1989, 1990 to 1999, 2000 to 2008), the nonparametric Kruskal-Wallis one-way ANOVA on ranks was used.

## Abbreviations

MC: microcystin; *mcy*: microcystin synthetase genes; NFQ: non-fluorescent quencher; PC-IGS: phycocyanin intergenic spacer; PCR: polymerase chain reaction; PSA: P700 apoprotein; qPCR: quantitative real-time polymerase chain reaction; SNP: single nucleotide polymorphism; TNA: TaqNuclease assay.

## Competing interests

The authors declare that they have no competing interests.

## Authors' contributions

VO performed the DNA extractions and qPCR measurements, analyzed the data, and drafted the manuscript. FS collected and archived all the samples from Lake Zürich. OK provided the data on phytoplankton and nutrients. RK designed the experiments, analyzed the data, and drafted the manuscript. All of the authors have read and approved the final manuscript.

## Supplementary Material

Additional file 1 **Additional file 1, Table S1 and Figures S1 to S3**. Supplementary file providing additional Table S1 and Figures S1 to S3 in one pdf file. Click here for file
