# Supplementary material for: Stability of toxin gene proportion in red-pigmented populations of the cyanobacterium Planktothrix during 29 years of re-oligotrophication of Lake Zürich
Source: BMC Biol. 2012 Dec 7;10:100. doi: 10.1186/1741-7007-10-100 (PMC3534634; doi:10.1186/1741-7007-10-100)
Supplement: Additional file 1 — , Table S1 and Figures S1 to S3. Supplementary file providing additional Table S1 and Figures S1 to S3 in one pdf file. [file 1741-7007-10-100-S1.DOC]

**Table S1.** Alignment of *Planktothrix* *mcy*T sequences of nontoxic strains (loss of 90% of the *mcy* gene cluster) and toxic strains (containing the *mcy* gene cluster) used for designing a Custom TaqMan SNP Genotyping Assay

| Strain | *mcy* gene cluster^a^ | Pigmentation^b^ | Lineage^c^ | Origin^d^ | Lake depth (m)^e^ | Sequence^f^ |
| --- | --- | --- | --- | --- | --- | --- |
| **Nontoxic strains** |  |  |  |  |  |  |
| No.2A | n | green | 1 | Markusbölefjärden (FI) | 9 | ACAGAGAAAGCCGAGTTGGTTTGTTCCC**A**CCAAGCTTCTATCTCCAAGGCGTTAGGTTTCAAATCT |
| No.41 | n | green | 1 | Jägerteich (AT) | 2 | ACAGAGAAAGCCGAGTTGGTTTGTT**T**CC**A**CCAAGCTTCTATCTCCAAGGCGTTAGGTTTCAAATCT |
| No.63, 66 | n | green | 1 | Jägerteich (AT) | 2 | ACAGAGAAAGCCGAGTTGGTTTGTTCCC**A**CCAAGCTTCTATCTCCAAGGCGTTAGGTTTCAAATCT |
| No.250, 251, 253, 254, 255, 256, 257 | n | green | 1 | Albufera Lagoon (ES) | 3 | ACAGAGAAAGCCGAGTTGGTTTGTTCCC**A**CCAAGCTTCTATCTCCAAGGCGTTAGGTTTCAAATCT |
| No.259, 263, 274, 281 | n | green | 1 | Wannsee (DE) | 9 | ACAGAGAAAGCCGAGTTGGTTTGTTCCC**A**CCAAGCTTCTATCTCCAAGGCGTTAGGTTTCAAATCT |
| No.299, 320 | n | green | 1 | Klinkenberger Plas (NL) | 30 | ACAGAGAAAGCCGAGTTGGTTTGTTCCC**A**CCAAGCTTCTATCTCCAAGGCGTTAGGTTTCAAATCT |
| No.307 | n | green | 1 | Klinkenberger Plas (NL) | 30 | ACAGAGAAAGCCGAGTTGGTT**C**GTTCCC**A**CCAAGCTTCTATCTCCAAGGCGTTAGGTTTCAAATCT |
| No.760, 788, 790 | n | green | 1 | St. Domingos (PT) | 42.5 | ACAGAGAAAGCCGAGTTGGTTTGTTCCC**A**CCAAGCTTCTATCTCCAAGGCGTTAGGTTTCAAATCT |
| No.781, 828, 829, 836, 837 | n | green | 1 | Nero (RU) | 4.7 | ACAGAGAAAGCCGAGTTGGTTTGTTCCC**A**CCAAGCTTCTATCTCCAAGGCGTTAGGTTTCAAATCT |
| PCC7805 | n | green | 1 | Veluwemeer (NL) | 5 | ACAGAGAAAGCCGAGTTGGTTTGTTCCC**A**CCAAGCTTCTATCTCCAAGGCGTTAGGTTTCAAATCT |
| PCC7811 | n | green | 1 | Paris, Vert le Petit (FR) | 2 | ACAGAGAAAGCCGAGTTGGTTTGTTCCC**A**CCAAGCTTCTATCTCCAAGGCGTTAGGTTTCAAATCT |
| PH22 | n | green | 1 | Bagsværd Sø (DK) | 3 | ACAGAGAAAGCCGAGTTGGTTTGTTCCC**A**CCAAGCTTCTATCTCCAAGGCGTTAGGTTTCAAATCT |
| SAG5.81 | n | green | 1 | Kiessee (DE) | 2 | ACAGAGAAAGCCGAGTTGGTTTGTTCCC**A**CCAAGCTTCTA**C**CTCCAAGGCGTTAGGTTTCAAATCT |
| No.277 | n | green | 1 | Wannsee (DE) | 9 | ACAGAGAAAGCCGAGTTGGTTTGTTCCC**A**CCAAGCTTCTATCTCCAAGGCGTTAGGTTTCAAATCT |
| No.364 | n | green | 1 | Moose Lake (CA) | 19.8 | ACAGAGAAAGCCGAGTTGGTTTGTTCCC**A**CCAAGCTTCTATCTCCAAGGCGTTAGGTTTCAAATCT |
| No.371, 372, 396, 552, 553, 557 | n | greenbrown | 1 | Moose Lake (CA) | 19.8 | ACAGAGAAAGCCGAGTTGGTTTGTTCCC**A**CCAAGCTTCTATCTCCAAGGCGTTAGGTTTCAAATCT |
| CCAP1459/15 | n | green | 1 | Lough Neagh (UK) | 34 | ACAGAGAAAGCCGAGTTGGTTTGTTCCC**A**CCAAGCTTCTATCTCCAAGGCGTTAGGTTTCAAATCT |
|  |  |  |  |  |  |  |
| **Toxic strains** |  |  |  |  |  |  |
| No.31/1, 32, 39 | y | green | 1 | Wannsee (DE) | 9 | ACAGAGAAAGCCGAGTTGGTTTGTTCCC**G**CCAAGCTTCTATCTCCAAGGCGTTAGGTTTCAAATCT |
| No.79 | y | green | 1 | Arresø (DK) | 40 | ACAGAGAAAGCCGAGTTGGTTTGTTCCC**G**CCAAGCTTCTATCTCCAAGGCGTTAGGTTTCAAATCT |
| SAG6.89 | y | green | 1 | Plußsee (DE) | 30 | ACAGAGAAAGCCGAGTTGGTTTGTTCCC**G**CCAAGCTTCTATCTCCAAGGCGTTAGGTTTCAAATCT |
| NIVA-CYA126/8 | y | green | 1 | Langsjön (FI) | 18 | ACAGAGAAAGCCGAGTTGGTTTGTTCCC**G**CCAAGCTTCTATCTCCAAGGCGTTAGGTTTCAAATCT |
| CCAP1459/11A | y | green | 2 | Windermere (UK) | 64 | ACAGAGAAAGCCGAGTTGGTTTGTTCCC**G**CCAAGCTTCTATCTCCAAGGCGTTAGGTTTCAAATCT |
| CCAP1459/14 | y | red | 2 | Loughrigg Tarn (UK) | 10.3 | ACAGAGAAAGCCGAGTTGGTTTGTTCCC**G**CCAAGCTTCTATCTCCAAGGCGTTAGGTTTCAAATCT |
| CCAP1459/16, CCAP1459/17 | y | green | 2 | Blelham Tarn (UK) | 15 | ACAGAGAAAGCCGAGTTGGTTTGTTCCC**G**CCAAGCTTCTATCTCCAAGGCGTTAGGTTTCAAATCT |
| CCAP1459/21 | y | green | 2 | Esthwaite Water (UK) | 16 | ACAGAGAAAGCCGAGTTGGTTTGTTCCC**G**CCAAGCTTCTATCTCCAAGGCGTTAGGTTTCAAATCT |
| CCAP1459/30 | y | red | 2 | Plöner See (DE) | 60 | ACAGAGAAAGCCGAGTTGGTTTGTTCCC**G**CCAAGCTTCTATCTCCAAGGCGTTAGGTTTCAAATCT |
| CCAP1459/31 | y | green | 2 | White Lough (UK) | 10.7 | ACAGAGAAAGCCGAGTTGGTTTGTTCCC**G**CCAAGCTTCTATCTCCAAGGCGTTAGGTTTCAAATCT |
| CCAP1460/5 | y | green | 2 | Kasumigaura (JP) | 10 | ACAGAGAAAGCCGAGTTGGTTTGTTCCC**G**CCAAGCTTCTATCTCCAAGGCGTTAGGTTTCAAATCT |
| No.3, 97, 111 | y | red | 2 | Mondsee (AT) | 68 | ACAGAGAAAGCCGAGTTGGTTTGTTCCC**G**CCAAGCTTCTATCTCCAAGGCGTTAGGTTTCAAATCT |
| No.21- | y | red | 2 | Figur (AT) | 12 | ACAGAGAAAGCCGAGTTGGTTTGTTCCC**G**CCAAGCTTCTATCTCCAAGGCGTTAGGTTTCAAATCT |
| No.40, 91/1, 110 | y* | red | 2 | Mondsee (AT) | 68 | ACAGAGAAAGCCGAGTTGGTTTGTTCCC**G**CCAAGCTTCTATCTCCAAGGCGTTAGGTTTCAAATCT |
| No.64 | y | red | 2 | Wörthersee (AT) | 86 | ACAGAGAAAGCCGAGTTGGTTTGTTCCC**G**CCAAGCTTCTATCTCCAAGGCGTTAGGTTTCAAATCT |
| No.80 | y | red | 2 | Schwarzensee (AT) | 54 | ACAGAGAAAGCCGAGTTGGTTTGTTCCC**G**CCAAGCTTCTATCTCCAAGGCGTTAGGTTTCAAATCT |
| No.108 | y | red | 2 | Irrsee (AT) | 32 | ACAGAGAAAGCCGAGTTGGTTTGTTCCC**G**CCAAGCTTCTATCTCCAAGGCGTTAGGTTTCAAATCT |
| No.139, 145, 161, 166, 169, 170, 178 | y* | red | 2 | Grabensee (AT) | 13 | ACAGAGAAAGCCGAGTTGGTTTGTTCCC**G**CCAAGCTTCTATCTCCAAGGCGTTAGGTTTCAAATCT |
| No.260 | y | green | 2 | Wannsee (DE) | 9 | ACAGAGAAAGCCGAGTTGGTTTGTTCCC**G**CCAAGCTTCTATCTCCAAGGCGTTAGGTTTCAAATCT |
| No.403, 405, 406, 496, 549, 550, 551 | y* | red | 2 | Moore (Crane) Lake (CA) | 26 | ACAGAGAAAGCCGAGTTGGTTTGTTCCC**G**CCAAGCTTCTATCTCCAAGGCGTTAGGTTTCAAATCT |
| No.761 | y | red | 2 | Reservoir Garcia (IT) | 43 | ACAGAGAAAGCCGAGTTGGTTTGTTCCC**G**CCAAGCTTCTATCTCCAAGGCGTTAGGTTTCAAATCT |
| No.775 | y | red | 2 | Reservoir Nicolletti (IT) | 36 | ACAGAGAAAGCCGAGTTGGTTTGTTCCC**G**CCAAGCTT**T~**ATCTCCAAGGCGTTAGGTTTCAAATCT |
| No.777, 778, 779 | y | red | 2 | Reservoir Nicolletti (IT) | 36 | ACAGAGAAAGCCGAGTTGGTTTGTTCCC**G**CCAAGCTTCTATCTCCAAGGCGTTAGGTTTCAAATCT |
| No.803, 808, 804, 805, 806, 807, 811 | y | green | 2 | Winnecook Lake (US) | 12.5 | ACAGAGAAAGCCGAGTTGGTTTGTTCCC**G**CCAAGCTTCTATCTCCAAGGCGTTAGGTTTCAAATCT |
| No.813, 814, 815 | y | green | 2 | China Lake (US) | 25.9 | ACAGAGAAAGCCGAGTTGGTTTGTTCCC**G**CCAAGCTTCTATCTCCAAGGCGTTAGGTTTCAAATCT |
| No.838, 840 | y | red | 2 | Reservoir Garcia (IT) | 43 | ACAGAGAAAGCCGAGTTGGTTTGTTCCC**G**CCAAGCTTCTATCTCCAAGGCGTTAGGTTTCAAATCT |
| PCC7821 | y | red | 2 | Gjersjøen (NO) | 64 | ACAGAGAAAGCCGAGTTGGTTTGTTCCC**G**CCAAGCTTCTATCTCCAAGGCGTTAGGTTTCAAATCT |
| CCAP1459/36 | y* | green | 2 | Gjersjøen (NO) | 64 | ACAGAGAAAGCCGAGTTGGTTTGTTCCC**G**CCAAGCTTCTATCTCCAAGGCGTTAGGTTTCAAATCT |
| No.758, 763, 764, 765, 766, 769, 770, 772 | y | green | 2 | Hormanjärvi (FI) | 21 | ACAGAGAAAGCCGAGTTGGTTTGTTCCC**G**CCAAGCTTCTATCTCCAAGGCGTTAGGTTTCAAATCT |
| No.822, 863, 865, 872, 873 | y | green | 2 | Pyhäjärvi (FI) | 35 | ACAGAGAAAGCCGAGTTGGTTTGTTCCC**G**CCAAGCTTCTATCTCCAAGGCGTTAGGTTTCAAATCT |
| No.67 | y* | red | 2 | Wörthersee (AT) | 86 | ACAGAGAAAGCCGAGTTGGTTTGTTCCC**G**CCAAGCTTCTATCTCCAAGGCGTTAGGTTTCAAATCT |
| No.82, 83/2 | y | red | 2 | Ammersee (DE) | 86 | ACAGAGAAAGCCGAGTTGGTTTGTTCCC**G**CCAAGCTTCTATCTCCAAGGCGTTAGGTTTCAAATCT |

^a^ presence/absence of the *mcy* gene cluster, a star indicates the inactivation of the *mcy* gene cluster by insertion or deletion [1]

^b^ as recorded under culture conditions (at 15°C and continuous light 5-10 µmol m^-2^ s^-1^, Osram Type L30W/77 Fluora), [1]

^c^ *Planktothrix* strains were assigned to two different lineages according to Christiansen et al [2]

^d^ Countries: FI = Finland, DE = Germany, DK = Denmark, UK = United Kingdom, AT = Austria, ES = Spain, NL = Netherlands, PT = Portugal, RU = Russia, FR = France, CA = Canada, JP = Japan, IT = Italy, US = USA, NO = Norway

^e^ maximum lake depth

^f^*P. agardhii* microcystin biosynthesis gene cluster (AJ441056.1, bp 552-617)

**Figure S1.** (A) Relationship between the total *Planktothrix* biovolume as determined by 16S rDNA, and the biovolume of the *Planktothrix* population determined by microscopical counting. (B) Relationship between the total *Planktothrix* biovolume determined by 16S rDNA and the biovolume of the same cells carrying the PC-IGS gene fragment. Biovolumes in mm^3^ L^-1^, symbols represent the mean values of four measurements per year and standard errors. For details on the regression curve see text.


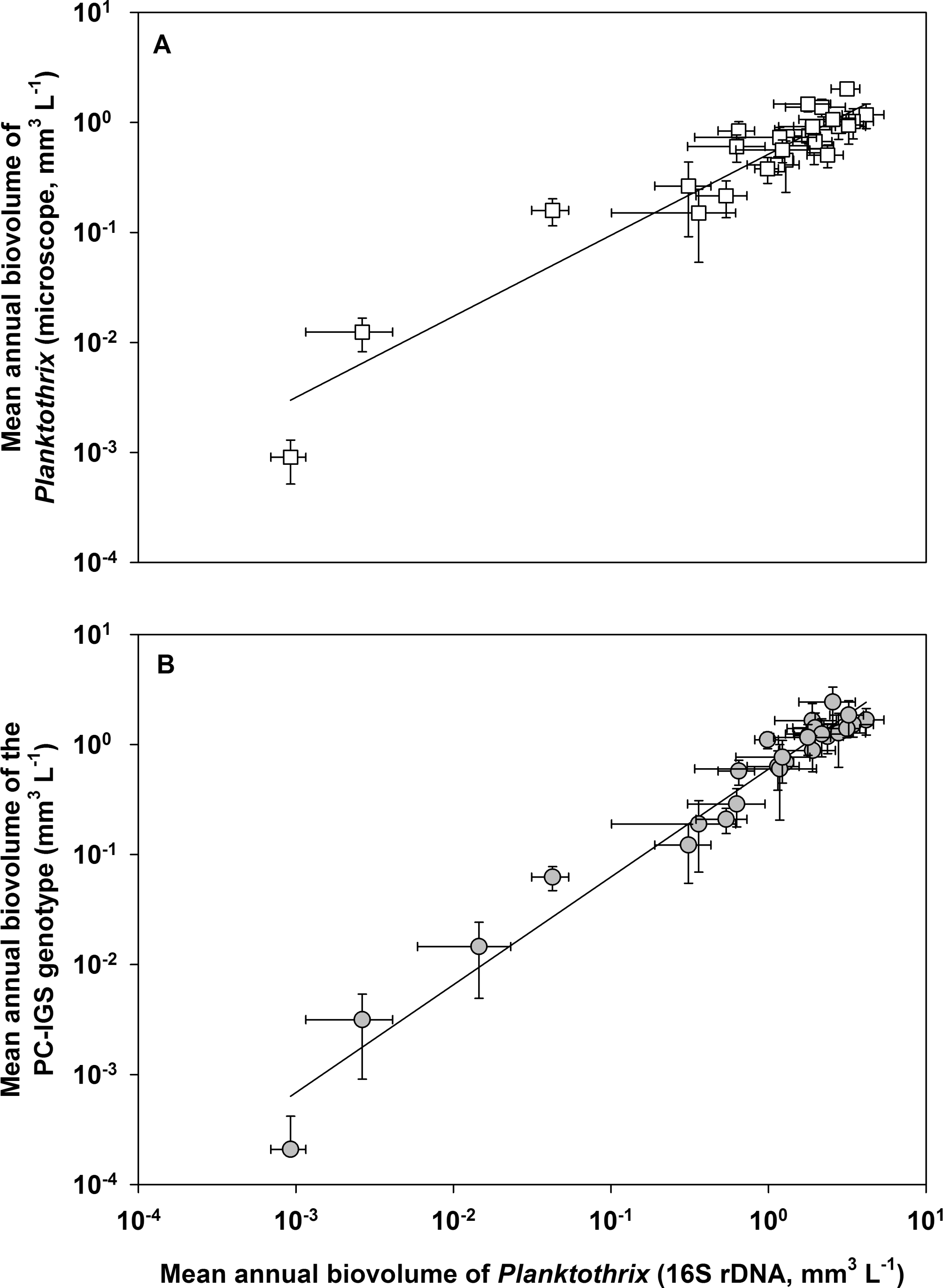


**Figure S2.** Allelic discrimination plot differentiating genotypes that lost (allele 1) or still contain (allele 2) the *mcy* gene cluster along the axis by a single nucleotide polymorphism within the *mcy*T gene. Serial dilutions of pure DNA of axenic strain PCC7811 (loss of the *mcy* gene cluster except of the *mcy*T gene), and PCC7821 (containing the *mcy* gene cluster) and mixtures of both strains containing 0.1, 0.2, 0.5, 1, 10, 20 and 50% of DNA of nontoxic strain PCC7811 (calculated as cell equivalents) are shown. When not visible, error bars are hidden behind the symbol.


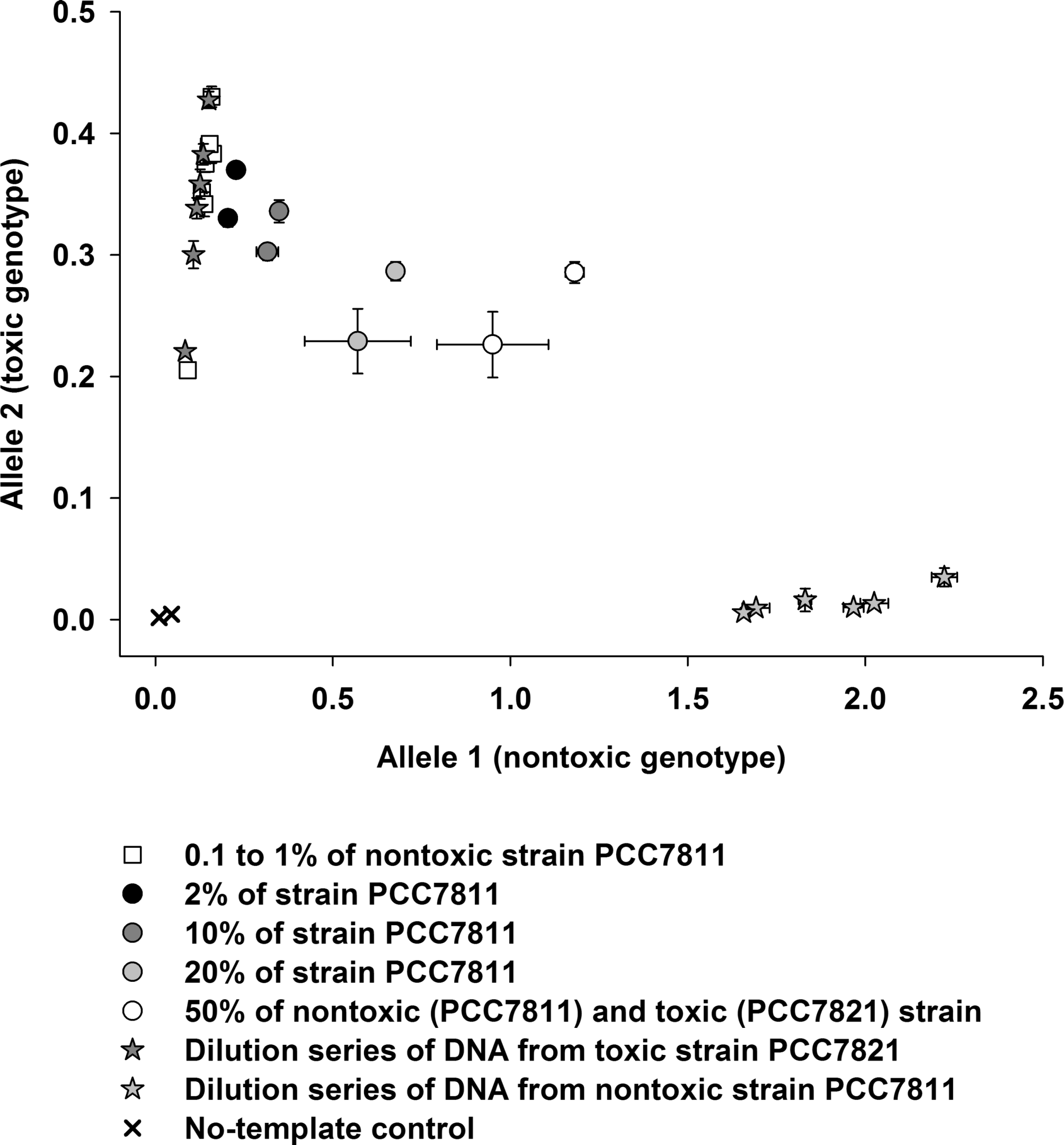


# Figure S3. Photograph of filters with phytoplankton harvested from different depths and stored dry at room temperature. The darker reddish color is indicative of the stratification of *Planktothrix* at a depth of 12 – 18m.


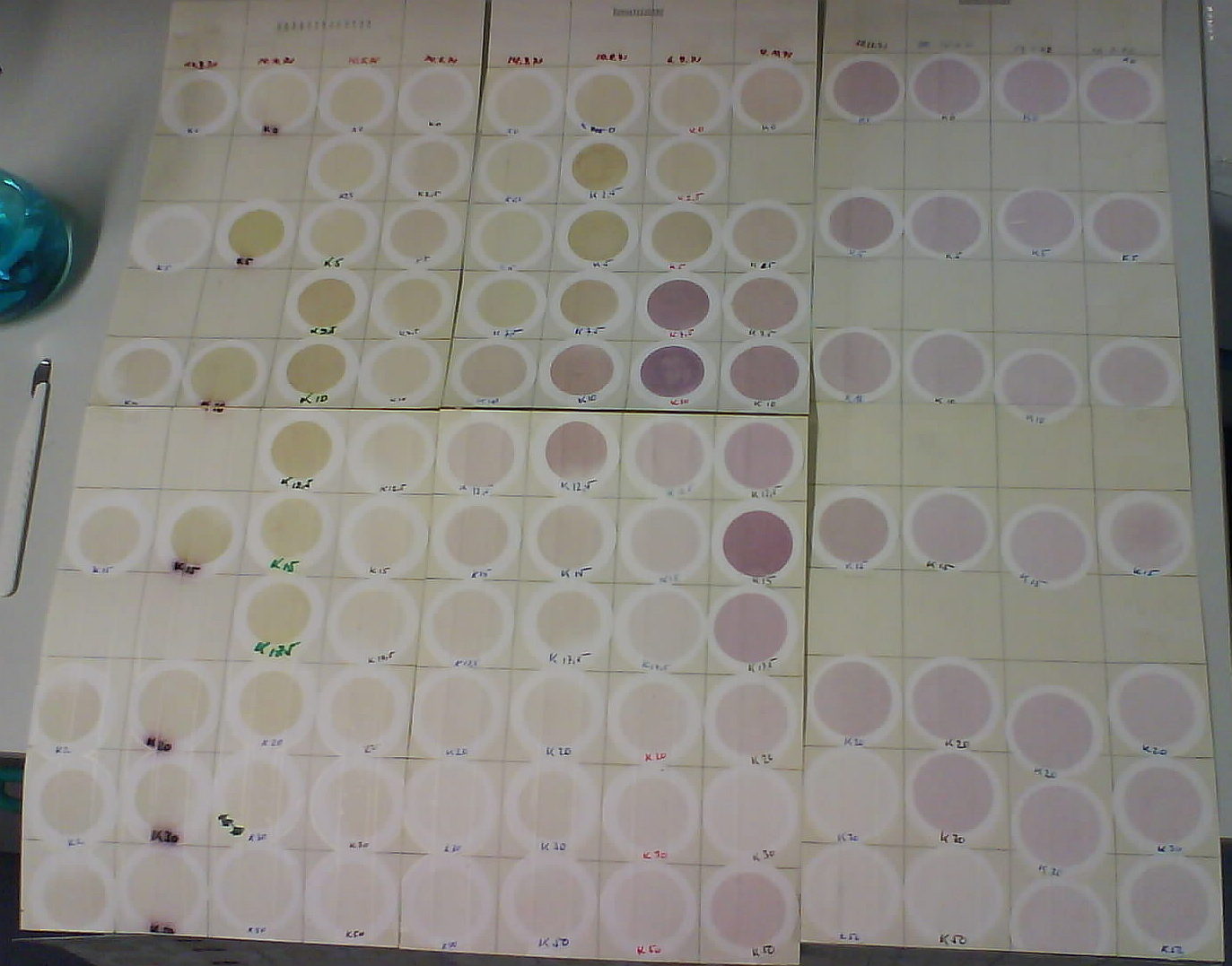


**References**

1. Kurmayer R, Christiansen G, Fastner J, Börner T: **Abundance of active and inactive microcystin genotypes in populations of the toxic cyanobacterium *Planktothrix* spp.** *Environ Microbiol* 2004, **6:**831-841.

2. Christiansen G, Molitor C, Philmus B, Kurmayer R: **Nontoxic strains of cyanobacteria are the result of major gene deletion events induced by a transposable element.** *Mol Biol Evol* 2008, **25:**1695-1704.
